# Clinical and Microbiological Outcomes of Implant-Supported Hybrid Abutment Crowns in the Posterior Region as a Function of Restorative Material: Exploratory Results of a 36-Month Randomized Clinical Trial

**DOI:** 10.3390/dj14100637

**Published:** 2026-10-04

**Authors:** Sigmar Schnutenhaus, Marla Weinmann, Constanze Olms, Jens Dreyhaupt, Andreas Rathke

**Affiliations:** 1 Center for Dentistry, Dr. Schnutenhaus Community Health Center (CHC) GmbH, Breiter Wasmen 10, 78247 Hilzingen, Germany; 2 Center of Dentistry, Department of Prosthetic Dentistry, University of Ulm, Albert-Einstein-Allee 11, 89081 Ulm, Germany; 3 Private Dental Practice of the Specialist Dentists Olms, Neuperverstraße 14, 29410 Salzwedel, Germany; 4 Institute of Epidemiology and Medical Biometry, University of Ulm, Schwabstraße 13, 89075 Ulm, Germany; 5 Faculty of Medicine, University of Ulm, 89081 Ulm, Germany; 6 Clinical Research, Dentsply Sirona, 78464 Konstanz, Germany

**Keywords:** hybrid abutment crown, dental implant, peri-implantitis, microbiome, marginal bone resorption, lithium disilicate, hybrid ceramic

## Abstract

**Background:** Hybrid abutment crowns combine a prefabricated titanium base with a custom, CAD/CAM-fabricated ceramic superstructure. Despite their increasing clinical use, only limited long-term in vivo data are available on how the restoration material influences clinical and microbiological outcomes. **Objective:** We investigated the clinical, radiographic, and microbiological outcomes of implant-supported hybrid abutment crowns in the posterior region over 36 months as a function of crown material. **Methods:** In a single-center, randomized, controlled clinical trial with blinded data analysis, 60 patients with a single-tooth gap in the posterior region received a Camlog iSy implant. Two implants were lost within the first four weeks, before prosthetic loading and randomization. The remaining 58 patients were randomized after stratification by sex and edentulous region to a polymer-infiltrated ceramic network hybrid ceramic (PICN; VITA Enamic, *n* = 28) or a lithium disilicate ceramic (LS2; IPS e.max CAD, *n* = 30). At 36 months, 57 patients (27 PICN, 30 LS2) were analyzed. The primary endpoint was implant survival; as no post-loading failure occurred, this was reported descriptively. Secondary endpoints were marginal bone level change (standardized intraoral radiographs, prosthetic baseline to 36 months), peri-implant probing depth (Florida Probe, baseline to 36 months; linear mixed-effects model), and the subgingival microbiome (micro-iDent plus, 6 and 36 months). Technical complications of this cohort have been reported separately 37. The trial was not powered to demonstrate equivalence, non-inferiority, or superiority. **Results:** The implant survival rate was 100%. Marginal bone resorption was low and stable. It did not differ significantly between crown materials (mesial mean difference 0.10 mm, 95% CI −0.11 to 0.31 mm; distal 0.15 mm, 95% CI −0.09 to 0.39 mm), nor by sex, jaw, implant length, diameter, or shoulder position. Probing depths remained low over 36 months without clinically relevant progression. The change in probing depth differed statistically between materials (mean difference 0.52 mm, 95% CI 0.12 to 0.92 mm; *p* = 0.012), but this difference was not considered clinically relevant. Microbiologically, most bacteria showed a stable to favorable course. For individual species (including *Fusobacterium nucleatum*, *Campylobacter rectus*, *Eikenella corrodens*, and *Capnocytophaga* spp.), deteriorations occurred more frequently, particularly in the LS2 group, without accompanying clinical or radiographic abnormalities. **Conclusions**: Within the limits of sample size and the 36-month follow-up, hybrid abutment crowns showed high peri-implant stability irrespective of crown material, with no clinically relevant differences between groups. This should not be interpreted as evidence of equivalence. When selecting materials, the higher technical complication rate in the PICN group reported separately for this cohort should also be considered.

## 1. Introduction

In recent decades, dental implants have emerged as an established and evidence-based treatment option for the rehabilitation of partially edentulous and edentulous patients, and can contribute significantly to the functional and psychosocial improvement in quality of life. By providing a stable, bone-anchored foundation, implants enable prosthetic restoration while preserving adjacent hard dental tissues, thereby ensuring the maintenance of oral structures [1]. Beyond osseointegration itself, factors such as fit accuracy, biological interactions, and the mechanical properties of the superstructure are particularly relevant to long-term prosthetic success and the survival rate of the entire implant restoration. Research in implant prosthetics therefore focuses on the further development of restorative materials in order to improve both biomechanical strength and esthetic outcomes. The introduction of digital CAD/CAM technology has fundamentally transformed the planning and fabrication of implant-supported restorations, enabling standardized yet highly precise manufacturing.

Hybrid abutment crowns, which combine an industrially manufactured titanium base with a custom, CAD/CAM-fabricated ceramic superstructure, are a particularly promising restoration concept [2]. A basic distinction is made between two-piece hybrid abutments with a separate crown and one-piece hybrid abutment crowns; in both variants, the titanium base is cemented extraorally onto the ceramic superstructure [3]. One-piece hybrid abutment crowns have only a single cement joint that can be cleaned extraorally under direct visualization, which helps prevent subgingival cement excess and is associated in the literature with high 1-year survival rates of up to 98.15% [3,4]. Current systematic reviews and meta-analyses describe overall favorable short- to medium-term clinical outcomes for the entire concept, with low rates of biological complications; however, they also point out the lack of reliable long-term data (≥5 years) as well as inconsistent study findings regarding the influence of the superstructure material [2,5]. Titanium-based abutment systems benefit from the mechanically robust, industrially standardized implant–abutment connection, while the customized ceramic crown can optimize peri-implant soft tissue integration and esthetic appearance [6,7]. The mechanical properties of the ceramics used vary considerably: Zirconia hybrid abutment crowns exhibit significantly higher fracture strength compared to one-piece zirconia abutments [6], while lithium disilicate ceramics are characterized by high translucency, good mechanical properties, and a connection technique to the titanium base that is technically simpler than that of zirconia [8].

More recently, polymer-infiltrated ceramic network (PICN) materials have been developed that combine ceramic and polymer components within a single matrix. Throughout this manuscript, we use the acronym PICN to denote this class of materials; HC is no longer used as a synonym. The PICN material used in the present study (VITA Enamic; VITA Zahnfabrik, Bad Säckingen, Germany) consists of a porous sintered feldspar ceramic matrix that is infiltrated with a polymer under pressure and heat, resulting in a dual network structure comprising 86 wt% ceramic and 14 wt% polymer [9,10]. Compared to monolithic glass-ceramics, this material is reported to have a lower modulus of elasticity, higher fracture toughness, and tribological properties that more closely resemble those of natural tooth enamel [10]; positive short- to medium-term clinical data are available for the commercially available product [11]. Lithium disilicate ceramic, by contrast, is an established, highly esthetic restorative material with proven mechanical stability, although technical complications such as fractures and chipping are discussed as the main risks [8].

In addition to the choice of material for the superstructure, the type of attachment used for implant-supported restorations is also important for peri-implant tissue preservation. Screw-retained and cement-retained restoration concepts exhibit different risk profiles. With cement-retained restorations, cement residues often remain subgingivally and are not completely removed; these are associated with peri-implant inflammation, biofilm formation, and progressive bone loss [12,13,14,15]. In contrast, screw-retained restorations tend to show more favorable values for probing bleeding and plaque index, but are technically more complex to fabricate and handle [12,16]. Since the one-piece hybrid abutment crowns examined in this study are both screw-retained and feature only a single cement joint that can be controlled extraorally, they potentially combine the advantages of both concepts.

The clinical success of implant-supported restorations is typically assessed based on implant survival as well as supplementary biological, technical, and esthetic parameters [15,17]. As early as 1986, Albrektsson defined five criteria for evaluating the long-term success of implants: clinical stability, the absence of peri-implant radiographic opacities, vertical bone loss of less than 0.2 mm per year after the first year of loading, the absence of signs of infection or inflammation, and a survival rate of at least 85% after 5 years and 80% after 10 years [18]. These criteria continue to serve as the minimum standard today, supplemented by prosthetic and esthetic assessment tools such as the Meijer Implant Crown Esthetic Index [19] and the Pink Esthetic Score [20]. For implant-supported single crowns, 5-year survival rates of approximately 97% are reported, whereby technical and biological complications together account for the majority of the reported events; the most frequent technical complications are loosening of the abutment screw, loss of crown retention, and chipping of the veneer ceramic [15].

Peri-implantitis is considered the greatest biological risk factor for the long-term success of dental implants. According to the consensus report of Working Group 4 of the 2017 World Workshop, peri-implant diseases are understood to be biofilm-associated, immune-mediated inflammatory processes; peri-implant health is characterized by the absence of bleeding on probing, swelling, and suppuration, whereas peri-implantitis is characterized by progressive radiographic bone loss [21]. Peri-implant mucositis, a reversible inflammation of the peri-implant mucosa without accompanying bone loss, serves as a precursor to peri-implantitis [22]. Systematic reviews and meta-analyses have shown a considerable range of variation in the reported prevalence of peri-implantitis, but have identified inadequate oral hygiene, lack of or irregular follow-up care, a history of periodontitis, and prosthetic factors such as the emergence profile and cleanability of the superstructure as key risk indicators [23,24,25,26,27].

Etiologically, peri-implantitis is understood as a biofilm-induced dysbiosis, characterized by a complex interaction between various microorganisms and the host immune system [28]. In peri-implant lesions, classic periodontopathogenic bacteria of Socransky’s red and orange complexes are frequently found, particularly *Porphyromonas gingivalis*, *Tannerella forsythia*, and Treponema denticola, as well as *Fusobacterium nucleatum* and *Prevotella intermedia* [29]. *Fusobacterium nucleatum* acts as a so-called bridge organism, promoting the coaggregation of numerous other bacterial species and thus facilitating biofilm maturation [30]; a recent meta-analysis revealed significantly elevated odds ratios for this bacterium, as well as for *Treponema denticola* and *Tannerella forsythia*, in association with peri-implant lesions [29]. In contrast, meta-analyses have failed to demonstrate a significant correlation between *Aggregatibacter actinomycetemcomitans* and peri-implant inflammation, and the pathogenic role of *Campylobacter rectus* also remains unclear despite frequent clinical detection [31]. Animal studies and clinical investigations also suggest that an increased peri-implant bacterial load is directly associated with the progression of marginal bone resorption, whereas remission is accompanied by lower microbial diversity [32,33].

PCR-based rapid testing methods are increasingly being used for the standardized assessment of subgingival bacterial load; compared to conventional anaerobic culture techniques, these methods are more time- and cost-efficient and are described as valid, with an overall agreement rate of 78–97% with culture methods [34]. The Florida Probe, an electronic constant-pressure probe developed in 1988, is increasingly being used to measure peri-implant probing depths; compared to manual probing methods, it offers higher reproducibility and measurement accuracy, and reduces examiner-dependent reading errors [35,36].

In addition to the biological evaluation of implant-supported restorations, the health economic perspective is also gaining increasing importance. Cost-effective implant systems with standardized prosthetic components, such as the iSy concept used in this study, represent an alternative to established, individually configured implant–abutment systems which currently lack reliable long-term clinical data. The combination of such a system with CAD/CAM-fabricated hybrid abutment crowns made of different restorative materials has so far been directly compared in only a few controlled clinical studies, although the posterior region in particular places special demands on materials and fabrication quality due to higher occlusal loads and limited visual control.

Against this background, the present clinical study investigates whether implant-supported hybrid abutment crowns in the posterior region differ—depending on the restoration material used (lithium disilicate ceramic versus hybrid ceramic)—in terms of implant survival, marginal bone contour, peri-implant probing depths, and subgingival microbiome over a period of 36 months. The null hypothesis assumed no significant material-dependent differences in the target parameters under investigation, while the alternative hypothesis exploratively assumed possible differences between the material groups.

## 2. Materials and Methods

### 2.1. Study Design

The present study was a single-center, randomized, controlled clinical trial with blinded data analysis. Randomization was performed by lot using sealed, opaque envelopes by an independent person not involved in the treatment to ensure concealed and unbiased group assignment. The primary endpoint was implant survival rate over 36 months post-implantation as a function of the material group; secondary endpoints included peri-implant probing depths, microbiological findings, and marginal bone contour. The study was approved by the Ethics Committee of the University of Ulm (file number 327/15, approval date 29 October 2015) and registered under DRKS ID DRKS00009628 (registration date 9 November 2015). Patients were recruited between [11/2015] and [03/2020]; the last 36-month follow-up examination was performed in [5/2024]. It was conducted in accordance with the Declaration of Helsinki; all participants provided written informed consent after receiving comprehensive information. The present manuscript reports the peri-implant radiographic, clinical and microbiological outcomes of the trial. The technical complications, the restoration-level outcomes and the primary radiographic analysis of the same cohort over three years have been published separately by our group [37]; to avoid redundant reporting, these outcomes are not repeated here and are referred to where relevant for interpretation. Because the two crown materials are visually distinguishable, neither the patients nor the treating clinician could be blinded to the allocation; the trial was therefore an open study with blinded outcome assessment and blinded statistical analysis. The following were blinded to allocation: (i) the examiner who measured the radiographs, who worked on anonymized images that were coded with study numbers only, presented in randomized order and, for the reliability subsample, without information on the time point; (ii) the external laboratory that performed the PCR analysis, which received coded samples; and (iii) the biostatistician (J.D.), who analyzed a coded data set with groups labeled A and B and was unblinded only after the analyses had been executed. The principal investigator (S.S.) treated the patients and participated in the interpretation of the data. The clinical probing measurements were performed by the treating investigator and were therefore not blinded; this is acknowledged as a limitation. Implant survival was defined as the primary endpoint when the protocol was written; since no failure occurred after prosthetic loading and both early failures occurred before randomization, this endpoint cannot discriminate between the materials, and the trial was not designed or powered to demonstrate equivalence, non-inferiority or superiority. Implant survival is therefore presented descriptively, and the continuous secondary outcomes are used to make informative material comparisons.

### 2.2. Patient Population

The study included patients with a single-tooth gap in the posterior region of the upper or lower jaw, whose extraction had occurred at least three months prior and whose gap was flanked by natural teeth or implants. Additional inclusion criteria were the presence of a natural reference tooth on the contralateral side, opposing dentition, as well as adequate oral hygiene and patient compliance. We excluded minors or individuals lacking legal capacity, patients with untreated acute periodontitis, heavy nicotine use (>10 cigarettes/day), use of bisphosphonates, pregnancy, alcohol or drug abuse, infectious diseases such as hepatitis B/C or HIV/AIDS, severe or uncontrolled diabetes mellitus, severe bruxism treated with a splint, planned immediate implantation, and clearly recognizable extensive need for bone augmentation (e.g., sinus lift). A total of 60 patients were enrolled in this study. Two patients were excluded within the first four weeks due to early implant loss prior to prosthetic loading, leaving 58 patients eligible for prosthetic loading and randomization. This baseline cohort comprised 35 women (60.3%) and 23 men (39.7%), with a mean age of 49.8 ± 11.9 years (range 21–75 years). Their baseline characteristics are summarized in Table 1 (Results).

Following randomization, 28 patients were assigned to Group 1 (hybrid ceramic, HC) and 30 to Group 2 (lithium disilicate ceramic, LS2). One further patient in Group 1 (HC) did not attend the 3-year follow-up examination and was therefore excluded from the final analysis. Consequently, the 3-year outcome evaluation was based on a total of 57 patients—27 in Group 1 (HC) and 30 in Group 2 (LS2). All patients underwent a complete periodontal examination before enrolment, and a periodontal diagnosis was established according to the 1999 classification valid at the time. Untreated periodontitis was an exclusion criterion, and patients with a history of periodontitis were included only after completed therapy with documented periodontal stability. From crown placement onwards, all patients were included in a structured, risk-adapted oral hygiene and maintenance program comprising individual oral hygiene instruction with demonstration of interdental and peri-implant cleaning, professional mechanical plaque removal at 6-month intervals, and re-instruction at every recall visit.

Baseline characteristics of the patient and implant cohorts—including gender distribution, age, implant site (jaw), implant length, and implant diameter—were recorded descriptively for the total cohort and for each material group (HC, LS2) and are presented in the Results section (Table 1).

### 2.3. Clinical Procedure

All patients were informed by the principal investigator (Prof. Dr. Sigmar Schnutenhaus, Center for Dentistry Prof. Dr. Schnutenhaus MVZ GmbH, Hilzingen) about the background, procedure, costs, and follow-up examinations. To plan the implant position and any necessary augmentation, a preoperative digital volume tomography scan with a voxel size of 0.2 mm was performed (Gendex CB500, Gendex Dental Systems, Des Plaines, IL, USA); virtual implant planning was carried out using SMOP software V 1.5.1 (Swissmeda, Zuerich, Switzerland) in at least two projection planes. When necessary (facial cortical bone thickness < 2 mm), augmentation was performed using particulate autogenous bone, a bone substitute material, and a collagen membrane. All patients received a Camlog iSy screw implant (ALTATEC GmbH, Wimsheim, Germany), which was placed according to the manufacturer’s instructions and allowed to heal open; the wound was closed with monofilament sutures for 7 days. After 2 months (good bone quality) or 3 months (cancellous bone quality), a closed impression was taken using a fast-setting vinyl polysiloxane (Imprint 4 Super Quick, 3M Espe, Seefeld, Germany) with custom trays; the master model was fabricated from scannable super-hard plaster (Henry Schein, Hong Kong, China).

### 2.4. Intervention Groups and Hybrid Abutment Crowns

In accordance with the predefined, stratified randomization, Group 1 (*n* = 28) received a one-piece hybrid abutment crown made of a polymer-infiltrated ceramic network hybrid ceramic (PICN; VITA Enamic, VITA Zahnfabrik, Bad Säckingen, Germany) consisting of 86 wt% ceramic and 14 wt% polymer [9,10], while Group 2 (*n* = 30) was treated with a monolithic lithium disilicate ceramic (LS2; IPS e.max CAD, Ivoclar Vivadent, Schaan, Liechtenstein), in which the entire supragingival restoration component, including the screw channel, was CAD/CAM-fabricated from a single ceramic block [8]. Both crown types were cemented extraorally onto a titanium abutment, screwed in place using the manufacturer’s recommended torque of 20 Ncm, and the screw channel was obturated with gutta-percha and a composite (Ceram-X mono, Dentsply De Trey, Bensheim, Germany).

### 2.5. Radiographic Examination

To assess marginal bone resorption, standardized intraoral single-view radiographs were taken for each patient at two time points—baseline (T1) and 36 months after implantation (T3). Measurements were performed using the validated image analysis software ImageJ (version 1.53); after individual calibration based on the known implant diameter, marginal bone loss was measured mesially and distally orthogonal to the implant shoulder, extending from its outer edge to the crestal bone margin. For each radiograph, two distance measurements and a control measurement of the implant diameter were performed; individual bone resorption was determined from the difference between T1 and T3. Comparable image-based analysis methods have also been successfully used in other split-mouth controlled studies of peri-implant bone structure for objective, examiner-independent quantification [38]. Standardization was achieved as follows: all radiographs were taken with the same intraoral sensor and film-holder system using the parallel (right-angle) technique, with an individualized occlusal bite registration attached to the holder for each implant site, so that sensor position, beam angulation and object-to-sensor distance were reproduced at T1 and T3; tube voltage, tube current and exposure time were kept constant. Images in which the implant shoulder or the mesial and distal bone crest were not clearly delineated, or in which the implant threads were not depicted symmetrically, were taken again. Residual differences in projection and magnification were compensated by calibrating every single radiograph to the known implant diameter, so that only calibrated ratios were evaluated. All measurements were performed by one examiner who was blinded to group allocation, on anonymized images presented in randomized order. Intra-examiner measurement precision was determined by measuring the same radiograph ten times. The mean measured distance was 1.312 mm mesially with a standard deviation of 0.047 mm and 0.985 mm distally with a standard deviation of 0.064 mm, corresponding to coefficients of variation of 3.58% mesially and 6.49% distally. The resulting 95% repeatability limit was 0.130 mm for the mesial and 0.177 mm for the distal measurement. Because this assessment was based on repeated measurements of a single radiograph, it quantifies the precision of the measurement procedure itself and does not include the additional variability introduced by repeated image acquisition; the repeatability limits therefore represent the lower bound of the measurement uncertainty against which the observed bone level changes have to be interpreted. The vertical position of the implant shoulder relative to the bone crest was determined on the calibrated baseline radiographs separately for the mesial and the distal aspect; because the crestal bone level frequently differed between the two aspects, four categories resulted: subcrestal and supracrestal on both aspects, and the two mixed categories in which the shoulder was subcrestal on one aspect and supracrestal on the other. These categories were recorded for all implants and were analyzed exploratively.

### 2.6. Performing of the Microbiological Analysis

The subgingival bacterial load was assessed at 6 and 36 months using the PCR-based micro-iDent plus test (Hain Lifescience, Nehren, Germany), which enables the semiquantitative detection of eleven potentially peri-implant pathogenic bacterial species: *Aggregatibacter actinomycetemcomitans* (Aa), *Porphyromonas gingivalis* (Pg), *Tannerella forsythia* (Tf), *Treponema denticola* (Td), *Prevotella intermedia* (Pi), *Parvimonas micra* (Pm), *Fusobacterium nucleatum* (Fn), *Campylobacter rectus* (Cr), *Eubacterium nodatum* (En), *Eikenella corrodens* (Ec), and *Capnocytophaga* spp. (Cs) [34]. Samples were collected at four defined sites (mesial, oral, distal, buccal) on both the implant crown and the contralateral natural reference tooth. Sampling followed a standardized protocol and was performed before any professional cleaning at that visit and before probing. Supragingival plaque was removed with sterile cotton pellets, and the site was dried and isolated with cotton rolls. Sterile paper points supplied with the test kit were inserted into the peri-implant or gingival sulcus at four defined sites (mesial, buccal, distal, oral) until slight resistance was felt and left in situ for 10 s. The four paper points of one unit were pooled in a single transport vial, so that one pooled sample was obtained per implant and one per contralateral reference tooth; consequently, the unit of analysis was the implant or the control tooth (one value per patient and unit, *n* = 57), no site-specific analyses were performed, and no clustering of sites had to be accounted for. Samples were shipped at ambient temperature on the day of collection and processed by the laboratory (Hain Lifescience, Nehren, Germany) blinded to group allocation. The test reports each species on an ordinal semiquantitative scale. For analysis, the change from baseline for each species was dichotomized into “improvement/stability” versus “deterioration,” separated by implant and control sides as well as by material group. The 6-month examination, i.e., the first examination after prosthetic loading, served as the microbiological baseline; the 36-month examination was the follow-up. For each species and each unit, the change in the ordinal semiquantitative score between these two time points was dichotomized: a decrease by at least one score category or an unchanged score was classified as “improvement/stability”, and an increase by at least one category as “deterioration”. Because units that were negative at both examinations necessarily fall into the “improvement/stability” category and thereby inflate it, this category is interpreted as the absence of a detectable increase rather than as microbiological improvement. A sensitivity analysis restricted to units that were positive at the microbiological baseline was not feasible, because the underlying semiquantitative results are not available in a form that permits re-analysis; this is addressed in the limitations. Analyses were stratified by unit (implant versus contralateral control tooth) and by material group; between-group proportions were compared with Fisher’s exact test and are reported with risk differences and 95% confidence intervals.

### 2.7. Probing Depths Measurement

Peri-implant probing depths were recorded at baseline and at 6, 12, 24, and 36 months using the electronic Florida Probe (Florida Probe Corporation, Gainesville, FL, USA) as a 6-point measurement on the implant and the contralateral control tooth. The Florida Probe is a constant-pressure probe with a hemispherical probe tip measuring 0.45 mm in diameter, which uses spiral springs to ensure a consistent probing pressure of 15 g and electronically records measurements with an accuracy of 0.2 mm, thereby minimizing examiner-dependent reading errors [35,36].

### 2.8. Statistics

Data were collected and managed using Microsoft Excel. Statistical calculations were performed using SAS (version 9.4) and SPSS (Version 29.0.2.0). Group comparisons for continuous variables were performed with the two-sample *t*-test (in case of two groups) or one-way ANOVA (in case of more than two groups). Associations between continuous variables were assessed using scatter plots and Pearson’s correlation coefficient. The percentage incl. 95% confidence interval (CI) was calculated to analyze the primary endpoint. Due to the exploratory character of this study and the lack of reliable preliminary work on comparable material combinations, a formal sample size calculation was not possible. All statistical tests were carried out at a two-sided significance level of alpha = 0.05. All results from statistical tests were interpreted as hypothesis-generating only and not as confirmatory due to the explorative nature of this study. No adjustment for multiple testing was made. The statistical analysis has been specified in more detail for this revision. Probing depth, which was recorded at baseline and at 6, 12, 24 and 36 months, was analyzed with a linear mixed-effects model including the fixed effects material group, time, and their interaction, and a random intercept per patient to account for the within-patient correlation of repeated measurements; an unstructured covariance matrix was used, and missing values were handled under the missing-at-random assumption (restricted maximum likelihood), so that all available observations contributed to the analysis. Adjusted mean differences between groups at each time point, and for the change from baseline, are reported with 95% confidence intervals. Marginal bone level change, which was available at two time points only, was compared between groups with the two-sample *t*-test and is likewise reported as a mean difference with a 95% confidence interval; one-sample *t*-tests were used to test whether changes differed from zero. Dichotomous microbiological outcomes were compared with Fisher’s exact test and are reported as risk differences with 95% confidence intervals. For all key between-group comparisons, effect estimates with 95% confidence intervals are given in addition to *p* values, and *p* values are reported in a uniform format. The exploratory subgroup analyses (gender, jaw, implant length, diameter, shoulder position, Ti-base height) were not prespecified as hypotheses, were not adjusted for multiplicity, and are reported as hypothesis-generating only. Because no formal sample size calculation was possible and no post-loading implant failure occurred, the trial has no power to demonstrate equivalence or non-inferiority; non-significant findings are therefore interpreted as an absence of evidence for a difference, not as evidence of absence of a difference.

## 3. Results

### 3.1. Patient Cohort and Implant Distribution

Of the 57 implants evaluated, 21 (36.8%) were placed in the maxilla and 36 (63.2%) in the mandible. The implant lengths used were predominantly 11 mm (54.4%), followed by 9 mm (40.4%) and 13 mm (5.3%); the most common diameters were 4.4 mm (42.1%), 3.8 mm (33.3%), and 5.0 mm (24.6%). The implant shoulder was positioned subcrestally in 60.8% of cases, supracrestally in 25.9%, and in a mixed position in 13.3% of cases. Between the two material groups (HC, *n* = 27; LS2, *n* = 30), gender, age, and implant-related parameters showed an even distribution (*p* > 0.05 in each case). Table 1 summarizes the key baseline characteristics of the patient and implant cohorts.

### 3.2. Implant Survival Rate

No implant loss was documented over the entire 36-month follow-up period following prosthetic restoration (implant survival rate: 100.0%, 95% CI: 93.7–100.0%); the implant survival rate was thus 100% in both material groups (Group 1 (HC): 100.0%, 95% CI: 87.2–100.0%; Group 2 (LS2): 100.0%, 95% CI: 88.4–100.0%). The only two implant failures of the trial occurred within the first four weeks after placement, i.e., before prosthetic loading and before randomization, and therefore cannot be attributed to either crown material. With zero post-loading events, the width of the confidence intervals shows that this endpoint cannot discriminate between the materials; it is reported descriptively and is not interpreted as evidence of comparable reliability of either material. Technical and prosthetic complications of the same cohort, which are the outcomes most likely to differ between the materials, are not reported here because they have been published separately [37]. In the previous analysis, crown fractures (25%), decementations (17.9%), and screw loosenings (14.3%) occurred in the PICN group, whereas in the LS2 group only screw loosening (12.5%) was observed. Implant survival and restoration success must therefore be clearly distinguished.

### 3.3. Marginal Bone Resorption

The measured changes in marginal bone level were generally within a narrow range, indicating high peri-implant stability. After 36 months, the mean marginal bone change was 0.10 ± 0.79 mm mesially and 0.13 ± 0.88 mm distally. These changes did not differ significantly from zero, either mesially (*p* = 0.340) or distally (*p* = 0.278).

Mean marginal bone level change was also compared between the sexes. Mesially, the mean change was −0.167 ± 1.017 mm in men and 0.277 ± 0.577 mm in women; the mean difference between men and women was −0.444 mm (95% CI −0.920 to 0.032 mm) and was not statistically significant (Welch *t*-test: *p* = 0.067). Distally, the mean change was 0.053 ± 1.260 mm in men and 0.211 ± 0.560 mm in women; the sex difference of −0.158 mm was likewise not statistically significant (95% CI −0.743 to 0.427 mm; *p* = 0.583). This comparison between the sexes was one of several exploratory subgroup analyses, was not prespecified as a hypothesis, and was not adjusted for multiplicity; it is therefore reported as hypothesis-generating only, and no effect of biological sex should be inferred from it. In the separate three-year analysis of the same cohort a statistically significant sex difference in mesial bone loss was reported [37], whereas in the present analysis, which is based on the Welch *t*-test and reports the corresponding confidence interval, the difference is not statistically significant. In the mandible, the mean bone change was 0.10 ± 0.93 mm mesially and 0.13 ± 0.99 mm distally, compared with 0.104 ± 0.47 mm mesially and 0.11 ± 0.64 mm distally in the maxilla. No statistically significant differences were found between the mandible and maxilla, either mesially (*p* = 0.971) or distally (*p* = 0.932).

There was also no significant difference between the two crown material groups, with the LS2 group exhibiting greater variability in values in both measurement directions. For HC crowns, the mean bone change was 0.09 ± 0.54 mm mesially and 0.12 ± 0.65 mm distally, while for LS2 it was 0.11 ± 0.97 mm mesially and 0.13 ± 1.06 mm distally. Differences between the two crown materials were not statistically significant, either mesially (*p* = 0.889) or distally (*p* = 0.952). Expressed as an effect estimate, the mean difference between the groups was 0.10 mm mesially (95% CI −0.11 to 0.31) and 0.15 mm distally (95% CI −0.09 to 0.39); the confidence intervals include differences that would be clinically irrelevant as well as the null value, so no conclusion of equivalence can be drawn [values to be inserted].

For a Ti-base height of 0.8 mm, the mean bone change was 0.04 ± 1.03 mm mesially and 0.02 ± 1.11 mm distally, whereas for a Ti-base height of 2.0 mm it was 0.15 ± 0.49 mm mesially and 0.22 ± 0.61 mm distally. Differences between Ti-base heights were not statistically significant, either mesially (*p* = 0.602) or distally (*p* = 0.417).

Regarding implant length, 9 mm implants showed a mean bone change of 0.25 ± 0.54 mm mesially and 0.33 ± 0.58 mm distally; 11 mm implants showed −0.03 ± 0.94 mm mesially and −0.06 ± 1.04 mm distally; and the three 13 mm implants showed 0.11; 0.17; 0.62 mm mesially and 0.29; 0.55; 0.79 mm distally. Overall, no statistically significant differences were found between implant lengths, either mesially (*p* = 0.374) or distally (*p* = 0.090). Notably, the 13 mm implant group included only three implants, so the corresponding means and standard deviations are only of limited interpretability.

For implant diameter, 3.8 mm implants showed a mean bone change of 0.12 ± 0.50 mm mesially and 0.04 ± 0.49 mm distally; 4.4 mm implants showed 0.09 ± 0.54 mm mesially and 0.24 ± 0.73 mm distally; and 5.0 mm implants showed 0.10 ± 1.32 mm mesially and 0.06 ± 1.38 mm distally. No statistically significant differences were found between diameter groups, either mesially (*p* = 0.985) or distally (*p* = 0.586).

With respect to implant position, subcrestal placement showed a mean bone change of 0.02 ± 0.45 mm mesially and 0.03 ± 0.57 mm distally, while supracrestal placement showed 0.43 ± 1.35 mm mesially and 0.31 ± 1.45 mm distally. In the “sub-/supracrestal” category, changes were −0.18 ± 0.64 mm mesially and 0.35 ± 0.82 mm distally, and in the “supra-/subcrestal” category they were 0.17 ± 0.04 mm mesially and −0.22 ± 0.27 mm distally. Comparison of means revealed no statistically significant differences between bone-level position categories, either mesially (*p* = 0.191) or distally (*p* = 0.516).

### 3.4. Probing Depths

At baseline, the mean probing depth (PD) at the implants was 1.488 ± 0.741 mm; after 36 months, it was 1.602 ± 0.697 mm (Δ = 0.115 ± 0.792 mm); the contralateral control teeth showed an average decrease of 0.348 ± 0.622 mm over the same period. In a comparison of the two groups, the change in PD at the implants was 0.388 ± 0.756 mm in Group 1 (HC) and −0.131 ± 0.752 mm in Group 2 (LS2); the adjusted mean difference between groups was 0.52 mm (95% CI 0.12 to 0.92; *p* = 0.012). Since the confidence interval lies entirely below the threshold of 1 mm that is generally regarded as clinically detectable, this difference is considered statistically significant but not clinically. Jaw position showed a significant influence on PD change at the implants in the total cohort (*p* = 0.010), although this effect did not reach significance within either material subgroup alone (HC: *p* = 0.057; LS2: *p* = 0.060). Implant diameter showed no significant overall influence (*p* = 0.191), but a significant effect was found within the HC group alone (*p* = 0.038); no such effect was observed in the LS2 group (*p* = 0.956). The influence of the implant shoulder position on the change in probing depth was not analyzed, because the two mixed shoulder position categories comprised only very few implants (see Section 3.1) and the resulting subgroups were too small for a meaningful comparison. With regard to implant length, the one-way ANOVA showed no significant difference in PD change at the contralateral control teeth overall (*p* = 0.112) or within the LS2 group (*p* = 0.597); however, a significant difference was found within the HC group alone (*p* = 0.017), a finding whose clinical relevance should be interpreted cautiously given the subgroup size. No significant length effect was detected at the implants themselves in any subgroup (overall: *p* = 0.088; HC: *p* = 0.486; LS2: *p* = 0.107).

Across all implants—irrespective of crown material or any other prosthetic or implant-related parameter—the correlation analysis revealed significant positive correlations between the mesial and distal PD changes (r = 0.309; *p* = 0.018) and between the distal PD change and the mesial bone level change (r = 0.317; *p* = 0.015). The strongest correlation was found between the mesial and distal bone level changes themselves (r = 0.837; *p* < 0.0001), indicating a predominantly symmetrical peri-implant reaction at both proximal measurement sites, independent of the restorative material used.

### 3.5. Microbiological Findings

The dichotomous evaluation of the eleven species detected by the micro-iDent plus test (improvement/stability versus deterioration compared to baseline) revealed a heterogeneous overall picture over 36 months. *Aggregatibacter actinomycetemcomitans* showed a consistently favorable trend. In the overall sample, 94.7% of the implant sites and 94.7% of the control tooth sites showed improvement or stability. *Prevotella intermedia* (91.2% implant, 84.2% control) and Eubacterium nodatum (83.9% implant, 82.5% control) also showed predominantly favorable trends. For *Porphyromonas gingivalis* (77.2% implant, 73.7% control) and *Treponema denticola* (73.7% implant, 68.4% control), the trend was also predominantly stable to declining, with the LS2 group tending to show slightly more favorable values than the HC group. *Tannerella forsythia* (50.9% implant and control) and *Parvimonas micra* (64.3% implant, 63.2% control) showed a roughly balanced to moderately heterogeneous ratio of improvement to deterioration, with no relevant differences between the groups.

In contrast, *Campylobacter rectus* (53.6% implant, 63.2% control), *Capnocytophaga* spp. (58.9% implant, 70.2% control), and, in particular, *Fusobacterium nucleatum* (51.8% implant, only 42.1% control) showed frequent deterioration. For *Eikenella corrodens*, the picture was clearly heterogeneous and varied depending on the material. In the overall sample, 58.9% of the implant measurement sites and 52.6% of the control tooth measurement sites showed improvement or stability. In Group 1 (HC), these proportions were 69.2% (implant) and 63.0% (control), respectively, while Group 2 (LS2) showed a numerically less favorable course, with rates of 50.0% (implant) versus 43.3% (control), respectively. However, a consistent, statistically significant material-dependent effect on the peri-implant microbiome as a whole could not be demonstrated, as the observed trends—including those for *Eikenella corrodens*—were not accompanied by a parallel deterioration of clinical or radiological parameters in the affected group.

Table 2 summarizes the dichotomized follow-up results (proportion of improved/stable measurement sites) for all eleven species examined on the implants. It must be emphasized that units which tested negative at both examinations are necessarily assigned to the category “improvement/stability”, so this category also contains units in which no target species was detected at any time. The reported proportions therefore reflect the absence of a detectable increase rather than a microbiological improvement, and they cannot be resolved further within the available, semiquantitatively scored data set. They should accordingly be regarded as descriptive and should not be interpreted as a measure of microbiological improvement.

Values indicate the percentage of measurement sites that showed improvement or no relevant deterioration compared to baseline findings; residual values correspond to the proportion of measurement sites that deteriorated.

In summary, implant survival, marginal bone distribution, and peri-implant probing depths over 36 months showed high stability with no significant differences between the two restorative materials; only gender was associated with bone distribution mesially. The microbiological findings were more heterogeneous, with sporadic, more frequent deterioration of individual species. This was most pronounced for *Eikenella corrodens* and *Fusobacterium nucleatum*, and tended to occur more frequently in the lithium disilicate group, but without accompanying clinical or radiological correlates.

## 4. Discussion

Implant-supported single-tooth restorations in the posterior region are considered an established treatment concept with a high success rate; however, despite a favorable prognosis, they are not free from the risk of biological and technical complications [15,16]. Peri-implant inflammation remains one of the greatest challenges to the long-term success of implant-supported prosthetic restorations and is closely associated with marginal bone loss [21]. According to the consensus of Working Group 4 of the 2017 World Workshop, peri-implant health cannot be defined based on a rigid radiographic threshold but must always be interpreted as a combination of clinical signs of inflammation, probing depth findings, and radiographic assessment [21,23]. This interpretive framework formed the basis for the evaluation of the present results.

### 4.1. Implant Survival and Marginal Bone Resorption

The 100% implant survival rate over 36 months lies at the upper end of the survival rates reported for implant-supported single crowns in systematic reviews [15,17]. With zero post-loading events in either group, however, this observation is compatible with a wide range of true failure rates and does not demonstrate comparative reliability of either crown material; it should be read as a descriptive result at the implant level only. The observed bone changes remained consistently within a very narrow range over 36 months, well below the threshold proposed by Albrektsson for physiological bone remodeling [18], and, together with the complete implant survival, indicate a high level of peri-implant stability for the restoration concept studied. The numerically greater mesial bone loss in women did not reach statistical significance (mean difference −0.444 mm, 95% CI −0.920 to 0.032 mm; *p* = 0.067) and should be interpreted with caution: the absolute differences are clinically minor, the confidence interval is wide and includes zero, and the greater variability observed in male patients suggests a possible influence of individual outliers. No sex-related effect on peri-implant bone level change can therefore be claimed from these data. In the literature, the influence of gender on peri-implant bone patterns has not been consistently demonstrated; current studies on prevention and risk factors instead emphasize plaque control, structured follow-up intervals, and comprehensive risk factor management as key determinants of peri-implant bone preservation [25,26]. No significant effects on bone distribution were found for jaw position, implant length, diameter, or shoulder position; this is consistent with a meta-analysis of 16 studies on subcrestal versus crestal implant shoulder position, which classifies the vertical positional difference itself as less decisive than the peri-implant soft tissue phenotype [39]. There was also no significant difference in bone contour between the two restorative materials; the slightly greater variation in the LS2 group should be interpreted as interindividual variability rather than a systematic material effect. This is consistent with the recent systematic review by Zhang et al., which describes overall favorable short- to medium-term clinical outcomes for hybrid abutment crowns with a low rate of biological complications, but at the same time highlights the lack of reliable long-term data (≥5 years), within which material-dependent effects might only become apparent [2].

### 4.2. Evaluation of the Microbiological Findings

Peri-implant diseases are now understood as biofilm-driven, host-mediated processes, whereby the clinical diagnosis is determined primarily by signs of inflammation, not by the detection of microorganisms alone [21]. The PCR-based micro-iDent plus test used in this study enables standardized, methodologically validated monitoring of defined marker bacteria [34], but does not cover the entire microbial ecology; current concepts view peri-implant dysbiosis as a functional shift in an entire microbial network rather than a phenomenon involving a single species [28]. The consistently favorable findings for *Aggregatibacter actinomycetemcomitans* are consistent with meta-analyses that found no significant correlation between this bacterium and peri-implant inflammation [31]. The inconsistent but generally favorable findings for the classic Red Complex species *Porphyromonas gingivalis* and *Treponema denticola*, as well as for *Tannerella forsythia*, are consistent with the literature, which indicates that while these bacteria are detectable with increased prevalence in peri-implant lesions, their presence alone is not necessarily synonymous with active peri-implant disease [28,29]. The frequent increases in *Fusobacterium nucleatum*, given its role as a bridge organism that promotes the coaggregation of other species, should be interpreted as a possible early indication of an incipient ecological shift in the biofilm, even in the absence of accompanying clinical symptoms [30]. The same applies to *Campylobacter rectus*, whose pathogenic role is described in the literature as unclear anyway [31], as well as to *Capnocytophaga* spp. and *Eikenella corrodens*. Their clinical significance appears to be highly context-dependent and is likely to manifest itself in interaction with other microorganisms rather than in isolation [28]. In particular, the pronounced group difference for *Eikenella corrodens* in favor of the hybrid ceramic group is noteworthy; however, given the number of species examined and the lack of correction for multiple testing, this should be interpreted as a hypothesis-generating finding rather than a confirmed one. Crucially, none of these microbiological deteriorations was accompanied by a parallel increase in clinical inflammatory parameters or progressive bone loss; thus, a direct association with active disease progression is not justified [21]. The slightly more favorable microbiological values in the HC group cannot be causally attributed to the restorative material in the absence of accompanying clinical differences; rather, the microbiological literature on active versus remission-phase courses suggests that prosthetic design, surface treatment, emergence profile, and cleanability are more significant mediating factors than the material itself [33].

### 4.3. Implant Survival, Biological Outcomes and Restoration Success

Implant survival and restoration success are not interchangeable: an osseointegrated, clinically stable implant may nevertheless carry a restoration that has fractured, become decemented, or required repair. In the present cohort, implant survival was 100% and the peri-implant biological outcomes were stable and comparable between the two materials, whereas the separate three-year analysis of the same patients [37] showed clearly different technical behavior, with crown fractures (25%), decementations (17.9%), and screw loosenings (14.3%) in the PICN group and only screw loosening (12.5%) in the LS2 group. The two levels of assessment must therefore be reported and interpreted separately: the biological equivalence suggested by the present data does not imply prosthetic equivalence, and the clinical recommendation for material selection is driven by the technical rather than the biological outcomes. Consequently, we can make no claim in the present manuscript regarding the prosthetic performance of the two materials; interested readers can refer to [37] for a discussion of that comparison.

### 4.4. Pocket Depths and Material Comparison

Peri-implant pocket depths remained low and stable over 36 months. Although the change in probing depth differed statistically significantly between the HC and LS2 groups (*p* = 0.012), the absolute difference was small and was considered clinically irrelevant, which indicates that no consistent association between the superstructure material and peri-implant pocket depth can be demonstrated [2,40]. This finding was consistent with the literature. Lithium disilicate has been established for years as a glass-ceramic material with high translucency and good mechanical properties [8], although the literature tends to focus on technical rather than biological complications, such as fracture and chipping [8]. Hybrid ceramics (PICN), by contrast, consist of a polymer-infiltrated ceramic network structure with mechanical and tribological properties that fall between those of traditional ceramics and resin-based materials [9,10]. Positive mid-term clinical data are available for the product used in this study [11]. Since the suprastructures examined were CAD/CAM-fabricated and—in the case of the one-piece hybrid abutment crown—were attached only at a single cement junction that could be visually inspected extraorally [3,4], a significant portion of the classic sources of error associated with subgingival restoration margins—such as excess cement or marginal irregularities—are eliminated; these issues are otherwise associated with plaque retention and peri-implant inflammatory reactions [13,14].

### 4.5. Clinical Implications

The current German S3 guideline on all-ceramic fixed implant-supported restorations explicitly addresses the choice of materials for implant prosthetic superstructures and, based on the current evidence, evaluates CAD/CAM-fabricated all-ceramic solutions—including lithium disilicate and hybrid ceramic materials—as fundamentally clinically suitable options for implant-supported single-tooth restorations based on the current evidence [41]. The results of the present study are consistent with this assessment: both materials tested demonstrated comparably high clinical, radiographic, and microbiological stability over 36 months, and the available data did not indicate a preference for either material. For dental practice, this implies that the choice between hybrid ceramic and lithium disilicate ceramic as a restorative material for hybrid abutment crowns in the posterior region should be considered secondary to consistent follow-up care and a biologically appropriate prosthetic design, and can instead be based on individual criteria such as esthetic requirements, risk of bruxism, or cost. Furthermore, both materials can be fully integrated into digital CAD/CAM workflows, which improves fabrication time, fit accuracy, and reproducibility compared to conventionally fabricated superstructures and thus potentially contributes indirectly to biological stability. In addition, recent consensus documents on the prevention of peri-implant diseases emphasize that a structured, risk-adapted follow-up plan—including regular professional cleaning and close clinical monitoring—makes a significant contribution to long-term peri-implant stability, regardless of the restoration material used [26,27].

### 4.6. Strengths and Limitations

The methodological strengths of this study include the randomized controlled study design; blinded data analysis; narrowly defined inclusion and exclusion criteria with the exclusion of known systemic risk factors; and the multimodal assessment of implant success based on radiological, clinical, and microbiological parameters using methodologically validated measurement instruments—the electronic Florida Probe for probing depths [35,36] and the PCR-based micro-iDent plus test for microbiological diagnostics [34]. However, several limitations must be emphasized. First, the trial was not designed to demonstrate equivalence or non-inferiority: with 27 versus 30 patients and no formal sample size calculation, the confidence intervals of all between-group comparisons are wide, and the absence of statistically significant differences must be interpreted as absence of evidence rather than as evidence of equivalence. Second, implant survival as the prespecified primary endpoint recorded no post-loading event and is largely independent of the superstructure; both implant failures occurred before randomization and thus outside the randomized comparison, so all material comparisons presented here are secondary and exploratory. Third, the technical complications and the restoration-level outcomes of this cohort are reported in a separate publication [37] and are not re-analyzed here. Readers must consult both reports for a complete picture, and the marginal bone level data of the two reports differ slightly because the present analysis is restricted to the 57 patients with complete microbiological follow-up. Fourth, bleeding on probing, suppuration and plaque scores were not part of the predefined data set of this trial, so the case definitions of the 2017 World Workshop [21] could not be applied in full; reference to that consensus is therefore restricted to its conceptual framework, and no peri-implant diagnoses were derived. Fifth, the microbiological outcome was analyzed as a dichotomized category, which by definition also contains units without any detection of the respective species at either examination; because the semiquantitative results are not available in a form that permits re-analysis, this category could not be resolved further and the microbiological findings are therefore reported descriptively only. Sixth, patients and the treating clinician could not be blinded because the materials are visually distinguishable, and the probing measurements were obtained unblinded, which may have introduced measurement bias; blinding was limited to the radiographic assessment, the microbiological laboratory and the statistical analysis. Seventh, the microbiological evaluation rests on a semiquantitative PCR panel of eleven marker species and on a dichotomized change score in which units negative at both examinations count as stable, and the first sampling took place 6 months after implant placement, so the early post-loading phase is not covered. Finally, no adjustment for multiple testing was made, and the single-center design with one experienced operator limits external validity. Limitations include the small sample size (58 randomized patients, 57 analyzed at 36 months), which in particular limits the statistical power of the subgroup and microbiome analyses and increases the risk of false-positive individual findings in the absence of correction for multiple testing, as well as the observation period limited to 36 months, which does not allow for a definitive assessment of 5- or 10-year outcomes.

### 4.7. Conclusion of the Discussion

No statistically significant or clinically relevant differences in the peri-implant radiographic, clinical and microbiological outcomes were detected between the two materials within the limits of the sample size and the 36-month follow-up. However, given the width of the confidence intervals, this must not be equated with proven equivalence, and the technical differences documented for the same cohort [37] point in the opposite direction at the restoration level. For clinical practice, this suggests that material selection should be based on the specific indication and implementation rather than on a general material recommendation; a biologically correct prosthetic design, a precise marginal fit, high surface quality, and a consistent aftercare and hygiene regimen appear to be crucial. Prospective studies with larger cohorts and longer follow-up periods are necessary, particularly to reliably assess the observed long-term microbiological trends.

## 5. Conclusions

Over 36 months, implant-supported single crowns in the posterior region made of PICN hybrid ceramic and of lithium disilicate ceramic both showed stable peri-implant conditions, and no statistically significant or clinically relevant difference in the radiographic, clinical and microbiological outcomes assessed here was detected between them. Because of the limited sample size (27 versus 30 patients) and the 36-month observation period, this absence of detected differences cannot be interpreted as evidence of equivalence of the two materials.

In clinical practice, material selection should therefore be based on the individual indication, occlusal load, available restorative space, esthetic requirements, and prosthetic workflow rather than on an assumed biological advantage, and it should take into account the technical complications documented for this cohort in the separate three-year report [37], which were more frequent with the PICN material. Prosthetic design, marginal adaptation, surface quality, maintenance, and oral hygiene were identical in both groups and were neither varied nor quantified as study variables; their relative contribution therefore cannot be derived from the present data and remains a hypothesis to be explored in dedicated prospective studies. The observed microbiological shifts, particularly involving *Eikenella corrodens* and *Fusobacterium nucleatum*, were not associated with clinical or radiographic signs of disease. Nevertheless, larger prospective studies with longer follow-up are needed to clarify their long-term relevance.

## Figures and Tables

**Table 1:** Baseline characteristics of the study population (*n* = 57).

Parameter	Total Cohort	Group 1 (HC, *n* = 27)	Group 2 (LS2, *n* = 30)
Gender, female	34 (59.6%)	17 (63.0%)	17 (56.7%)
Gender, male	23 (40.4%)	10 (37.0%)	13 (43.3%)
Age, years (mean ± SD)	50.2 ± 12.0	51.4 ± 11.2	49.2 ± 12.8
Implant site: upper jaw	21 (36.8%)	10 (37.0%)	11 (36.7%)
Lower jaw implant region	36 (63.2%)	17 (63.0%)	19 (63.3%)
Implant length 9 mm	23 (40.4%)	10 (37.0%)	13 (43.3%)
Implant length 11 mm	31 (54.4%)	14 (51.9%)	17 (56.7%)
Implant length 13 mm	3 (5.3%)	3 (11.1%)	0 (0.0%)
Implant diameter 3.8 mm	19 (33.3%)	10 (37.0%)	9 (30.0%)
Implant diameter 4.4 mm	24 (42.1%)	13 (48.1%)	11 (36.7%)
Implant diameter 5.0 mm	14 (24.6%)	4 (14.8%)	10 (33.3%)

**Table 2:** Proportion of improved/stable measurement sites per species after 36 months (implant side).

Species	Total Cohort	Group 1 (HC)	Group 2 (LS2)
*Aggregatibacter actinomycetemcomitans* (Aa)	94.7%	88.9%	100%
*Prevotella intermedia* (Pi)	91.2%	92.6%	90.0%
*Eubacterium nodatum* (En)	83.9%	84.6%	83.3%
*Porphyromonas gingivalis* (Pg)	77.2%	74.1%	80.0%
*Treponema denticola* (Td)	73.7%	70.4%	76.7%
*Parvimonas micra* (Pm)	64.3%	53.8%	73.3%
*Capnocytophaga* spp. (Cs)	58.9%	76.9%	43.3%
*Eikenella corrodens* (Ec)	58.9%	69.2%	50.0%
*Campylobacter rectus* (Cr)	53.6%	65.4%	43.3%
*Fusobacterium nucleatum* (Fn)	51.8%	57.7%	46.7%
*Tannerella forsythia* (Tf)	50.9%	51.8%	50.0%

## Data Availability

The complete documentation of all the patients enrolled in this study belongs to the authors and is available only upon reasonable request.
